# Supplementary material for: The Quinazoline Otaplimastat (SP-8203) Reduces the Hemorrhagic Transformation and Mortality Aggravated after Delayed rtPA-Induced Thrombolysis in Cerebral Ischemia
Source: Int J Mol Sci. 2022 Jan 26;23(3):1403. doi: 10.3390/ijms23031403 (PMC8835804; doi:10.3390/ijms23031403)
Supplement: Supplementary file 1 [file ijms-23-01403-s001.zip › ijms-1452614-supplementary.pdf]

Supplementary information

Supplementary figures and figure Legends

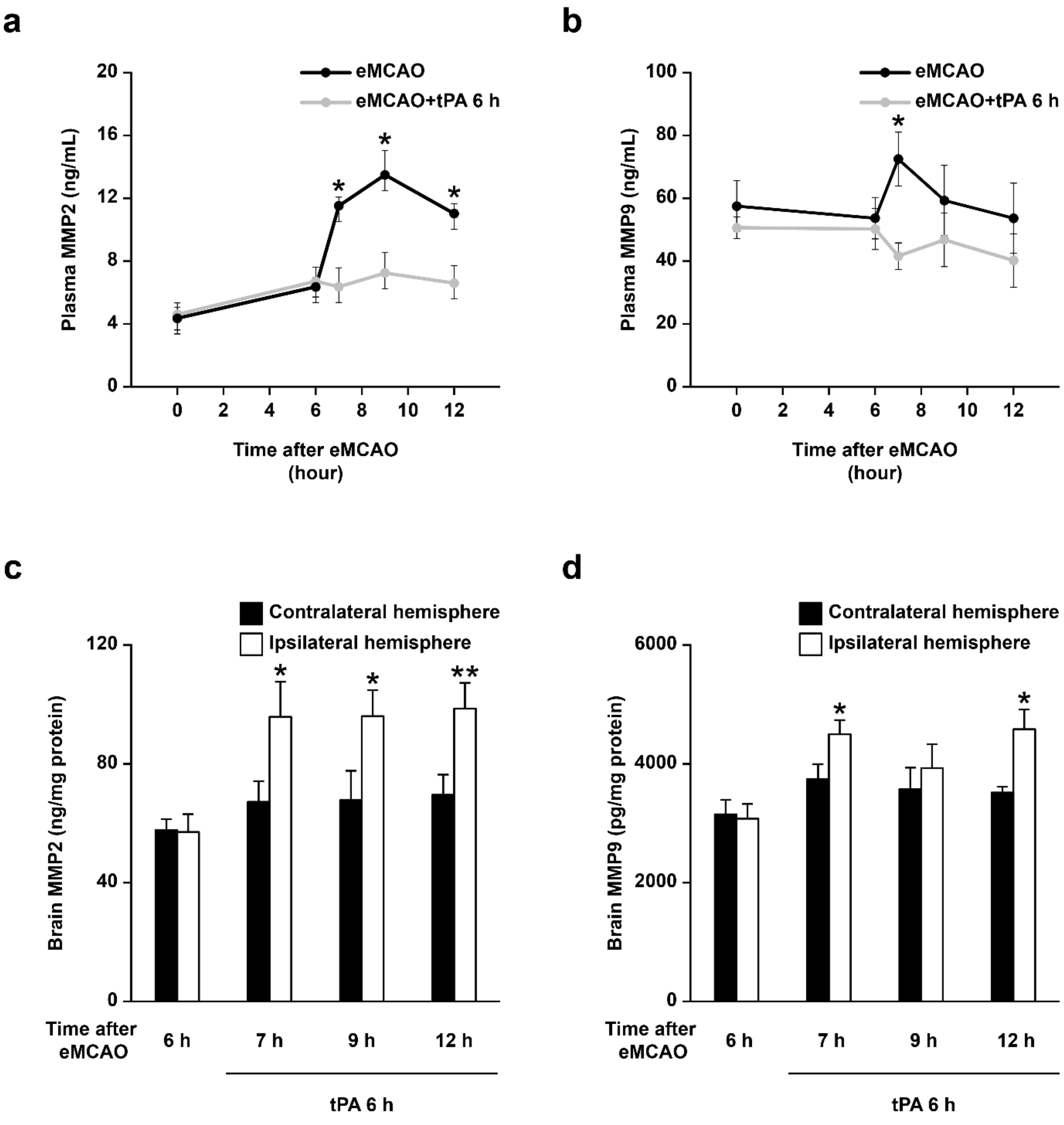

Supplementary Figure S1. Delayed rtPA treatment up-regulates MMP2/9 protein expression in plasma and brain tissue after embolic ischemia. Time-course of MMP2/9 levels in plasma (a-b) and brain lysates (c-d) after delayed rtPA treatment in an embolic stroke model. rtPA (10 mg/kg) was administered 6 h after the onset of ischemia and levels of MMP2 and 9 determined at the indicated time-points. Data are expressed as mean ± S.D. Statistical differences were analyzed with the Kruskal-Wallis followed by Mann-Whitney test (*n*=6-10, *p<0.05, compared to the non-rtPA treated group).

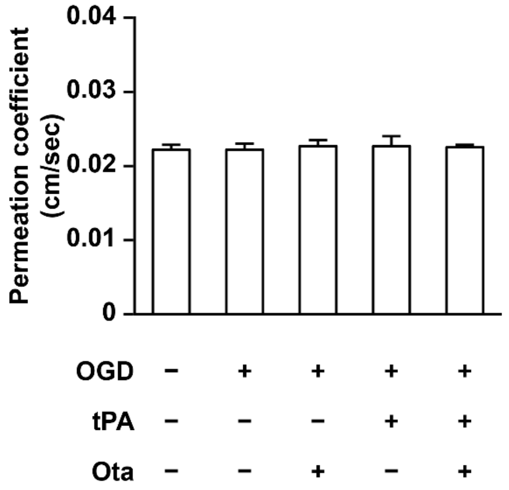

Supplementary Figure S2. Single-cultured endothelia permeability is not affected by OGD/tPA exposure.

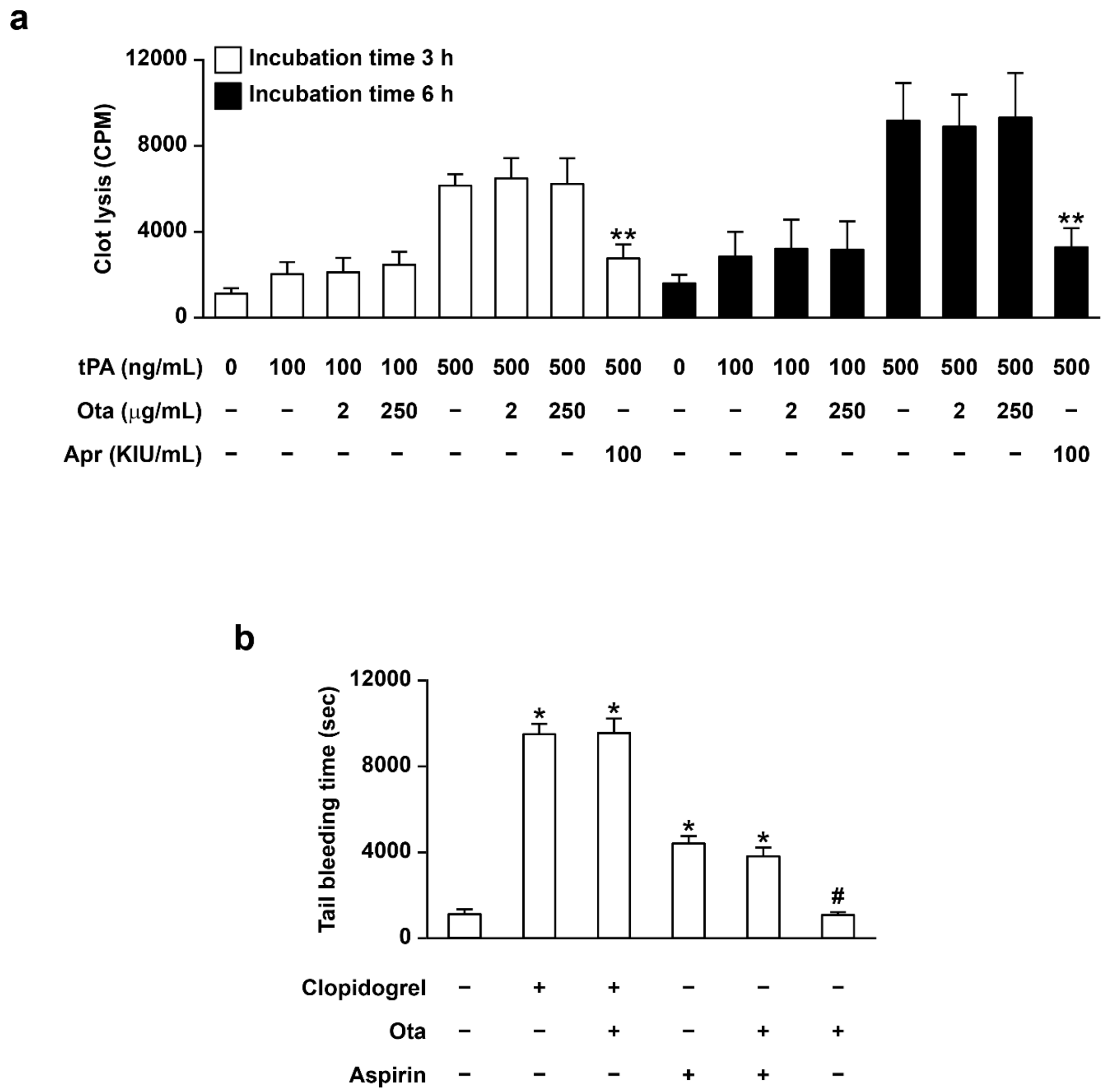

Supplementary Figure S3. **Otaplimastat does not affect thrombolytic activity of rtPA and antithrombotic effect of antiplatelet agents**. (a) Effect of otaplimastat(Ota) on clot lysis, with aprotinin (Apr) used as a positive control drug. Data are expressed as means ± SD of 10 independent experiments (**p<0.01, compared to rtPA alone). (b) Effect of otaplimastat on tail bleeding. Data are expressed as means ± S.D. and analyzed for statistical significance with Kruskal-Wallis followed by Mann-Whitney test. *n*=6 (*p<0.05, compared to control).

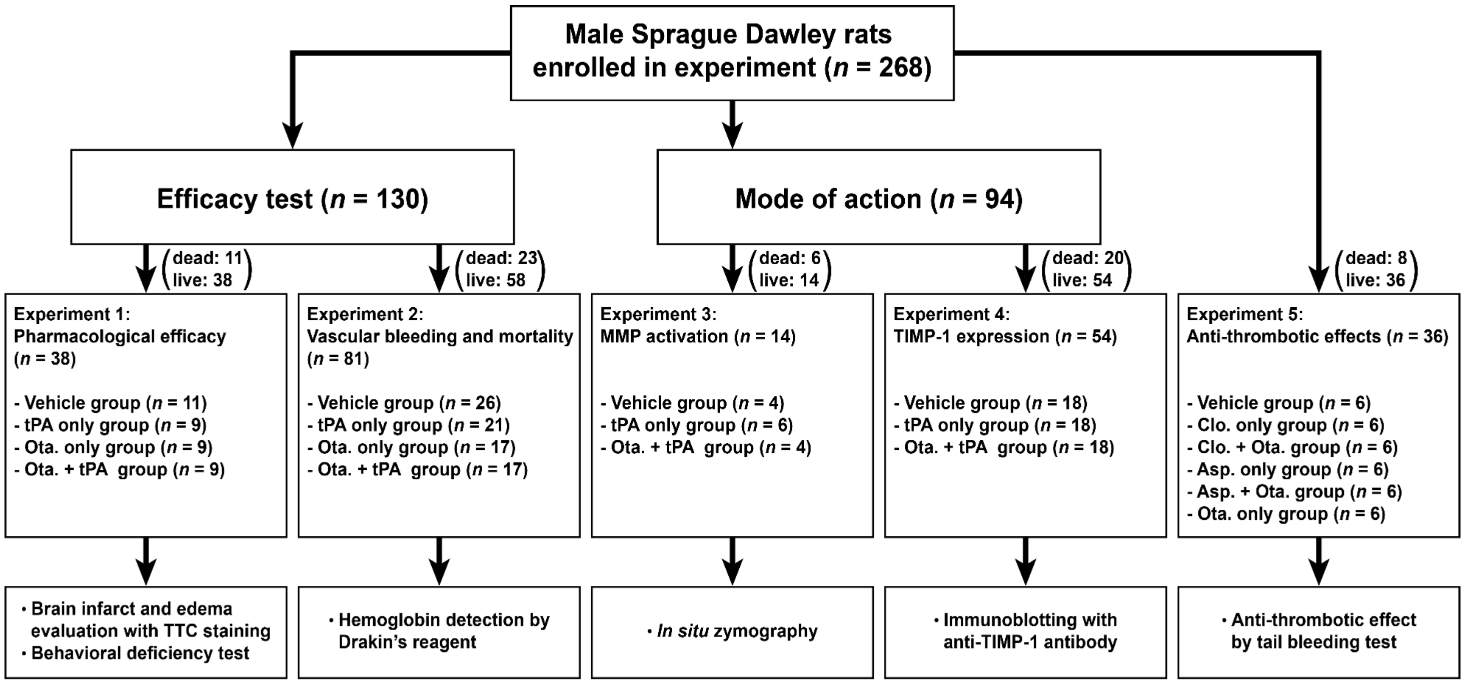

Note: Ota., Otaplimastat; Clo., Clopidogrel; Asp., Aspirin

Supplementary Figure S4. **Flowchart of animal experiments.** As shown in Fig. 1f, the mortality rate measured at 24 hours after eMCAO is 5~45% depending on the drug treatment. All tests except the mortality test were conducted on live animals.

Supplementary Table S1. Physiological variables for combined treatment of Otaplimastat (Ota) and tPA

**Paramete**	Rectal Temperature (°C)	pH	Glucose (mg/dL)	PaO2 (mmHg)	PaCO2 (mmHg)	MaBP (mmHg)
Before embolism						
Vehicle	36.87 ± 0.16	7.40±0.02	145.50±20.42	137.83±10.74	45.12±2.73	110.50±5.13
tPA	36.97±0.26	7.40±0.05	133.71±37.68	135.14±9.81	45.16±5.15	106.29±6.26
Ota+tPA	36.88±0.10	7.42±0.03	148.00±26.61	139.83±15.87	42.73±2.54	107.83±6.37
5.5 h after embolism						
Vehicle	38.13±0.55	7.40±0.05	147.00±32.92	135.50±19.87	43.62±5.00	123.00±5.02
tPA	38.29±0.40	7.43±0.02	150.43±9.91	143.57±15.87	37.96±4.92	125.14±5.15
Ota+tPA	37.93±0.23	7.40±0.03	139.67±21.21	140.67±12.04	44.90±5.48	121.17±8.06
7 h after embolism						
Vehicle	37.58±0.24	7.44±0.02	136.83±18.68	142.83±10.87	40.47±3.44	119.67±0.82
tPA	37.80±0.79	7.41±0.04	143.71±17.42	149.14±16.81	39.40±5.75	108.57±11.98
Ota+tPA	37.48±0.45	7.40±0.03	136.17±20.23	151.80±7.16	44.95±6.56	115.83±6.68

Physiological variables were measured 1 h after treatment with otaplimastat and rtPA (MABP, mean arterial blood pressure; PaO_2, partial arterial pressure oxygen; $PaCO_2$, partial arterial pressure of CO_2). Values are presented as means ± S.D (*n*=6-7).

Supplementary material & methods

Physiological variables

A catheter was inserted into the rat left femoral artery for monitoring arterial blood pressure and periodic blood collection. Physiological variables were measured 15 min before embolism and 1 h after otaplimastat and rtPA administration (5.5 h and 7 h after embolism, respectively). Body temperature was monitored with a rectal thermometer. Blood pH, glucose content, arterial partial O_2 pressure (PaO_2) and arterial partial CO_2 pressure ($PaCO_2$) were assessed using iSTAT® CG8+ cartridges with an automated pH/blood gas analyzer (iSTAT, Abott Lab., IL, USA). Mean arterial blood pressure (MABP) was measured using a DigiMed blood pressure analyzer (Micro-Med, Louisville, KY, USA).

Clot lysis analysis

The *in vitro* clot lysis assay was performed as reported previously (Colucci et al., 1993). Briefly, 2 mL donor blood was mixed with 20 μL ^{125}I-fibrinogen (400,000 cpm), $CaCl_2$ (0.0125 M, final concentration) and thrombin (1 U/mL), followed by incubation at 37℃ for 1 h. Clots generated were cut into pieces 10 mm in length and washed in saline at room temperature. Each clot was transferred to 1.5 mL tubes and incubated with rtPA, aprotinin or otaplimastat in 1 mL autologous plasma at 37°C. Next, the supernatant was transferred to a radioimmunoassay (RIA) tube and radioactivity measured with a γ-counter (Wallac 1480 WIZARD3; Perkin Elmer, Waltham, MA, USA). The degree of lysis was evaluated after 3 and 6 h of incubation by counting radioactivity.

Tail transection bleeding test

In clinical circumstances, patients are often treated with antiplatelet agents, such as aspirin and clopidogrel. Accordingly, to evaluate the possible interactions between antiplatelet agents and otaplimastat, the tail transection bleeding test was performed as reported previously (Berry et al., 1994; Ryu et al., 2009). Briefly, animals were fasted for 12 h and antiplatelet agents (30 mg/kg clopidogrel or 100 mg/kg aspirin) administered orally followed by sequential intravenous administration of otaplimastat (6 mg/kg). The tail was cut 4 mm from the tip using a surgical blade to induce bleeding (hemorrhage) and hemostasis time subsequently measured.

Supplementary References

Berry,C., Girard, D., Lochot, S., and Lecoffre, C. 1994. Antithrombotic actions of argatroban in rat models of venous, 'mixed' and arterial thrombosis, and its effects on the tail transection bleeding time. Br. J. Pharmacol. 113, 1209-1214.

Colucci, M., Cavallo, L.G., Agnelli, G., Mele, A., Burgi, R., Heim, J., et al. 1993. Properties of chimeric (tissue-type/urokinase-type) plasminogen activators obtained by fusion at the plasmin cleavage site. Thromb. Haemost. 69, 466-472.

Ryu, K.H., Han, H.Y., Lee, S.Y., Jeon, S.D., Im, G.J., Lee, B.Y., et al. 2009. *Ginkgo biloba* extract enhances antiplatelet and antithrombotic effects of cilostazol without prolongation of bleeding time. Thromb. Res. 124, 328-334.
